# The Functional Significance of Black-Pigmented Leaves: Photosynthesis, Photoprotection and Productivity in *Ophiopogon planiscapus* ‘Nigrescens’

**DOI:** 10.1371/journal.pone.0067850

**Published:** 2013-06-24

**Authors:** Jean-Hugues B. Hatier, Michael J. Clearwater, Kevin S. Gould

**Affiliations:** 1 School of Biological Sciences, University of Auckland, Auckland, New Zealand; 2 Department of Biological Sciences, University of Waikato, Hamilton, New Zealand; 3 School of Biological Sciences, Victoria University of Wellington, Wellington, New Zealand; University of Hyderabad, India

## Abstract

Black pigmented leaves are common among horticultural cultivars, yet are extremely rare across natural plant populations. We hypothesised that black pigmentation would disadvantage a plant by reducing photosynthesis and therefore shoot productivity, but that this trait might also confer protective benefits by shielding chloroplasts against photo-oxidative stress. CO_2_ assimilation, chlorophyll *a* fluorescence, shoot biomass, and pigment concentrations were compared for near isogenic green- and black-leafed *Ophiopogon* *planiscapus* ‘Nigrescens’. The black leaves had lower maximum CO_2_ assimilation rates, higher light saturation points and higher quantum efficiencies of photosystem II (PSII) than green leaves. Under saturating light, PSII photochemistry was inactivated less and recovered more completely in the black leaves. In full sunlight, green plants branched more abundantly and accumulated shoot biomass quicker than the black plants; in the shade, productivities of the two morphs were comparable. The data indicate a light-screening, photoprotective role of foliar anthocyanins. However, limitations to photosynthetic carbon assimilation are relatively small, insufficient to explain the natural scarcity of black-leafed plants.

## Introduction

The leaves of black mondo grass, *Ophiopogon* *planiscapus* Nakai ‘Nigrescens’, are exceptionally dark. Indeed, their chromaticity coordinates approach those of a flat black paint commonly used as the standard in colour comparisons, and are comparable to those of black-pigmented fruits from various plant species [1,2]. Black pigmentation is common among berries and seed coats, and it occurs occasionally in the sterile organs of some flowers [3,4]. However, black-pigmented leaves are exceedingly rare in nature, prominent only among certain genera of mosses, such as *Andreaea* and *Grimmia*, and of liverworts such as *Cephalomitrion*, *Isophyllaria*, and *Marsupella* [5,6,7,8,9,10,11]. There are no reports of natural communities of vascular plants with black leaves. Although many of the ornamental angiosperms currently popular for landscaping possess black or dark purple leaves [12], these arose either from the selection and propagation of sports, or from directed breeding programmes.

The dearth of black-leafed vascular plants is an intriguing problem. It may simply be that blackness does not confer any selective advantage to plants. Alternatively, dark coloured foliage could present a physiological handicap. It was previously postulated that the black pigmentation would restrict the transmission of light from the leaf epidermis to underlying chlorenchyma, thereby compromising photosynthesis efficiency and reducing shoot productivity [1]. In a comparison of the profiles of light penetration through leaves from near-isogenic lines of black- and green-leafed *O* *. planiscapus* ‘Nigrescens’, the spongy mesophyll cells of black leaves were found to receive less light in total than did those of green leaves. However, only green light was abated; the profiles of transmission of red and blue light were identical for the green and black leaves [1]. Green light is undoubtedly an important energy source that can be used in photosynthesis especially by the abaxial mesophyll, but its contribution to the total carbon assimilation is substantially smaller than that from either red or blue light [13,14,15]. It is possible, therefore, that black pigments do not appreciably impair leaf photosynthesis. In the absence of published data on the photosynthesis of black leaves, the relationship between black pigmentation and productivity remains far from certain.

Black pigmentation might also benefit plants under certain environmental conditions. The black colour of *O* *. planiscapus* ‘Nigrescens’ is achieved through luxuriant concentrations of both anthocyanins and chlorophylls in the leaf mesophyll [1]. Substantial experimental evidence accrued over recent years indicates that foliar anthocyanins can protect photosynthetic cells from the adverse effects of excess light quanta (reviewed in 16,17,18,19). When chloroplasts receive more quanta than they can use in photosynthesis, they show a sharp decline in the quantum efficiency of photosystem II (PSII). Protracted exposures to strong light, particularly in combination with another stressor such as drought or low temperatures, can lead to the production of free radicals that potentially damage thylakoid membranes and photosystem proteins [13,20]. Anthocyanins, by intercepting a proportion of the photons that might otherwise cause damage, have the potential to reduce the incidence, and mitigate the severity, of photo-oxidative assault [21]. It follows that a superabundance of anthocyanins, such as those in the leaves of *O* *. planiscapus* ‘Nigrescens’, might be particularly beneficial for plants in exposed locations that experience climatic extremes. The black coloration might further assist cellular metabolism by converting a portion of the absorbed light energy into heat, thereby increasing leaf temperatures in a cold environment. Interestingly, the black-leafed ecotypes of many bryophytes reside in Antarctica or Alaska, areas with strong insolation, high UV–B levels, and low temperatures [5,22].

To date, no study has explicitly addressed the possible benefits or disadvantages of black pigmentation in leaves. Here, carbon assimilation, shoot productivity, chlorophyll *a* fluorescence, and propensities for photoinhibition are compared in near-isogenic lines of green- and black-leafed *O* *. planiscapus* ‘Nigrescens’, an herbaceous perennial native to Japan. We hypothesise that under full sunlight, black pigmentation would disadvantage leaves by intercepting quanta that might otherwise be used for photosynthesis. Under light-limiting conditions, however, black pigmentation may confer an advantage by protecting shade-acclimated chloroplasts from the effects of photo-oxidative stress.

## Materials and Methods

### Plant growth conditions

**Figure 1:**
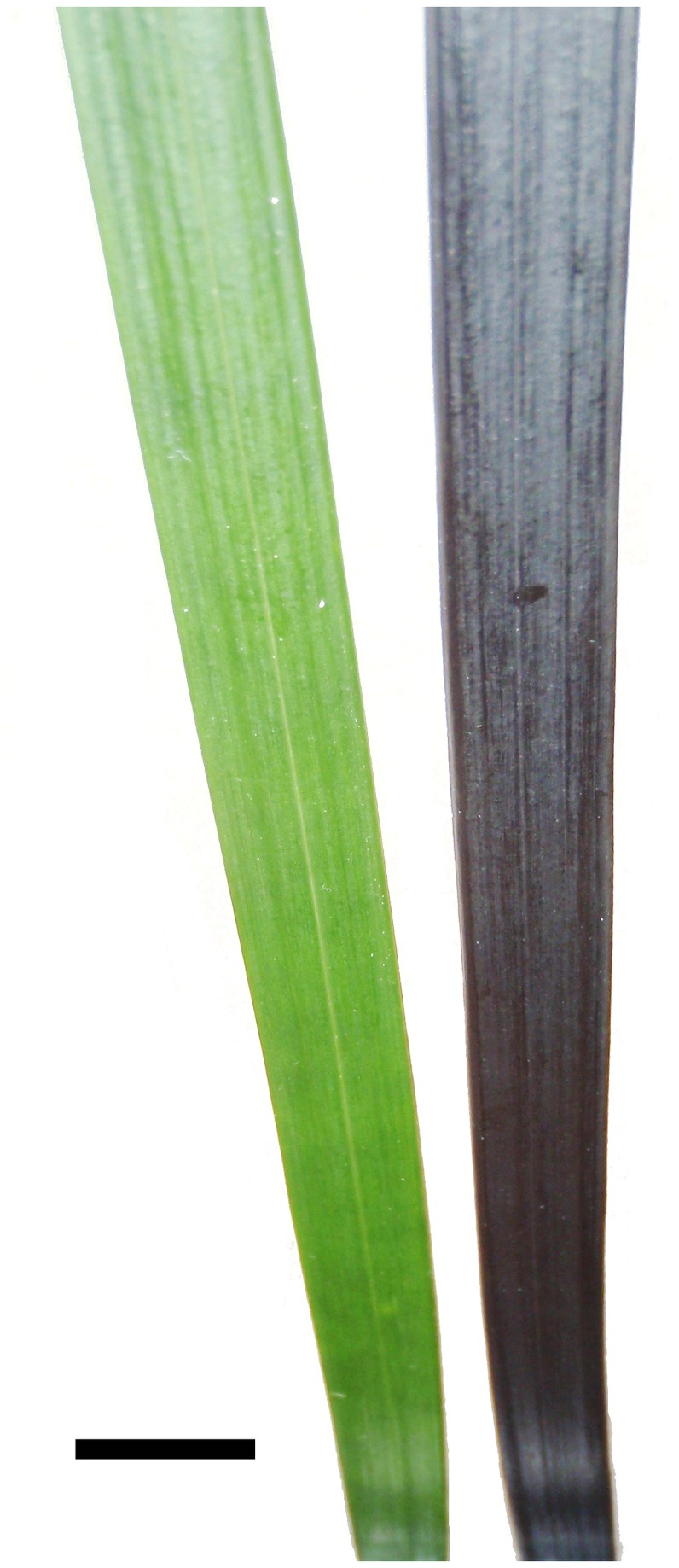
Photograph of portions of green and black leaves of *Ophiopogon* *planiscapus* ‘Nigrescens’. Bar = 5 mm.

Black- and green-leafed phenotypes of *Ophiopogon* *planiscapus* ‘Nigrescens’ Nakai (Figure 1) were obtained from an Auckland nursery. The two morphs were considered near-isogenic; the green phenotype had arisen spontaneously in approx. 20% of the progeny from crosses among the black plants, which were themselves clonal. Individual shoots were separated from each plant, re-potted, and randomly distributed over a 50m^2^ plot on the University of Auckland grounds. From September 2003 to February 2004, the plants were divided equally across three light treatments: 100%, 40% or 10% full sunlight. The different light regimes were obtained by covering plants with one or two layers of shade cloth; plants growing under the 100% sunlight regime were not covered. The spectral composition of light incident on the adaxial leaf surfaces of plants in each light regime was determined at 1 pm, when the sun was at its zenith, using a LI1800 spectroradiometer (Li-Cor Instruments, Lincoln, NE, USA) at scanning intervals of 2 nm over a 350-1100 nm bandwidth. All plants received a ratio of red (600-700 nm) to blue (400-500) wavebands of 1.15 ± 0.05, and a ratio of red to far red light (660: 730 nm) of 1.17 ± 0.04.

### Pigment quantification

The second youngest fully-expanded leaf from each of ten plants per treatment was randomly collected from both phenotypes. Foliar chlorophylls and carotenoids were extracted in 80% acetone on ice. Concentrations of these pigments were estimated by spectrophotometry from optical densities obtained with a Shimadzu dual beam UV-1601 spectrophotometer, using the equations provided by Lichtenthaler [23].

Anthocyanins were extracted in acidified methanol (MeOH: H_2_O:HOAc at 11:5:1 v/v/v) and measured spectrophotometrically using the following equation:

$${\text{Absorbance}}_{\text{Anthocyanin}}={\text{Amax}}_{\text{535nm}}-\text{0}{\text{.24A}}_{\text{653nm}}$$ [24]

Absorbances were converted to µmol per g fresh weight using a molar extinction coefficient of 33,000 mol^-1^ cm^-1^ [25]. Concentrations of UV-A and UV–B absorbing compounds were estimated from the same acidified methanolic extracts as absorbances at 360 nm and 280 nm, respectively.

### CO_2_ assimilation

The photosynthetic responses of leaves to light and CO_2_ were recorded using a portable photosynthesis system equipped with an LED light source (LI6400 and 6400-02B, LICOR, Nebraska). Light and CO_2_ response curves were recorded for 8 plants of each colour morph grown under full sunlight (the same leaves were used for the two types of response curve). Two to three leaves were positioned across the system cuvette and allowed to reach maximum stomatal conductance and rates of photosynthesis at 1500 µmol m^-2^ s^-1^ PAR before starting measurements. Leaf temperature was held at 20°C, and the vapour pressure deficit was between 0.8 and 1.4 kPa. For CO_2_ response curves, leaves were held under saturating irradiance at 1500 µmol m^-2^ s^-1^. Light response curves were fitted with a non-rectangular hyperbola [26], and the parameters A_max_ (light-saturated rate of photosynthesis), ϕ_a_ (apparent quantum efficiency), R_d_ (dark respiration) and Θ (convexity) estimated by non-linear regression (Photosyn Assistant, Dundee Scientific, Dundee, U.K.). Stomatal conductance at light saturation (g_s max_) was recorded as the average stomatal conductance when irradiance ≥ 1000 µmol m^-2^ s^-1^. The light saturation point (Q_sat_) was estimated as the irradiance required for modelled assimilation to reach 85% of A_max_, and the light compensation point (Q_comp_) as the irradiance when modelled photosynthesis was zero. Plots of photosynthesis as a function of intercellular CO_2_ concentration (c_i_) were fitted with the mechanistic model of von Caemmerer and Farquhar [27] and the parameters J_max_ (electron transport rate at saturating c_i_) and V_cmax_ (maximum rate of carboxylation) estimated by non-linear regression.

### Chlorophyll *a* fluorescence

Light response curves for chlorophyll *a* fluorescence were obtained for the adaxial surfaces of ten green and ten black leaves of *O* *. planiscapus* ‘Nigrescens’ that had been growing outdoors under full sunlight. Measurements were done on the fourth youngest fully expanded leaf from randomly selected plants. All measurements were recorded in a temperature-controlled incubator at 5° or 20°C using a Hansatech FMS2 pulse amplitude modulated fluorescence monitoring system (Hansatech Instruments, Ltd., UK). Actinic light was supplied by a halogen lamp (Osram, 8V-20W) via a fibre optic cable. Plants were dark acclimated for 1 h, and then minimum and maximum chlorophyll fluorescence parameters (F_o_ and F_m_, respectively) determined. Quantum yields of photosystem II (Ф_PSII_) were recorded after irradiating leaves with white light at 40, 270, 950, 1300, 1500 and 2000 µmol photon m^-2^ s^-1^ for 20 min each. Ф_PSII_ was estimated as:

$${\text{ΔF/F}}_{\text{m}}\text{’ = }\left({\text{F}}_{\text{m}}\text{’}-{\text{F}}_{\text{t}}\right){\text{/F}}_{\text{m}}\text{’}$$

where F_t_ = steady state fluorescence under actinic light prior to a saturating flash, and F_m_’ = maximum fluorescence following a saturating flash [28]. Photochemical quenching (qP) and non-photochemical quenching (NPQ) coefficients were calculated as described by Maxwell and Johnson [28].

#### Light treatments

The chlorophyll fluorescence of both leaf phenotypes was monitored before, during and after a saturating light treatment. Sun-grown plants were acclimated for 1 h in the dark at 5° or 20°C, and then the maximum quantum efficiencies for PSII (F_v_/F_m_) were determined, where F_v_=F_m_-F_o_. The plants were then irradiated with white light supplied by the Hansatech FMS2 at 1500 µmol photon m^-2^ s^-1^ for 30 min, during which ΔF/F_m_’, qP, and NPQ values were recorded. They were returned to darkness, and F_v_/F_m_ measurements recorded at regular intervals over 60 min.

#### Diurnal changes in chlorophyll a fluorescence

Plants that had acclimated under high, medium or low light flux were transferred to full sunlight on 10 February 2004 (late summer). Chlorophyll a fluorescence was measured on the adaxial surface of the fourth-youngest fully expanded leaf from five green and five black plants. Values of F_o,_ F_m_, and of ambient and leaf temperatures were determined for dark-acclimated plants pre-dawn. From 8 am, and every half hour thereafter until 7 pm (sunset), values of F_t_, F_m_’, F_o_’ and leaf temperature were recorded. To measure adaxial leaf temperatures, the thermocouple included in the FMS2 chlorophyll fluorescence system was shaded. Measurements of PAR incident at the leaf surface and ambient temperature were recorded using a data logger (Delta-T Devices, Cambridge, UK). This experiment was repeated two days later.

#### Productivity

Commencing February 2004, the total growth of shoots over a 150-day-period was recorded for 10 green and 10 black plants growing in each light regime. For each plant, increases in numbers of leaves and shoots per plant, in total leaf area and in shoot dry weight were determined.

#### Statistics

Differences between green and black plants in productivity, chlorophyll *a* fluorescence values and in the concentrations of chlorophylls, carotenoids and anthocyanins were tested by factorial ANOVA using SPSS 14.0 for Windows. Prior to assessment, values were tested for normality (Kolmogorov-Smirnov test) and homoscedasticity (*F*_max_ test). If the factorial ANOVA revealed significant effects, a Tukey honestly significant difference post hoc test (*p* < 0.05) was performed to group homogeneous means.

#### Data availability

All data are freely available on request.

## Results

### Photosynthesis and chlorophyll *a* fluorescence

**Figure 2:**
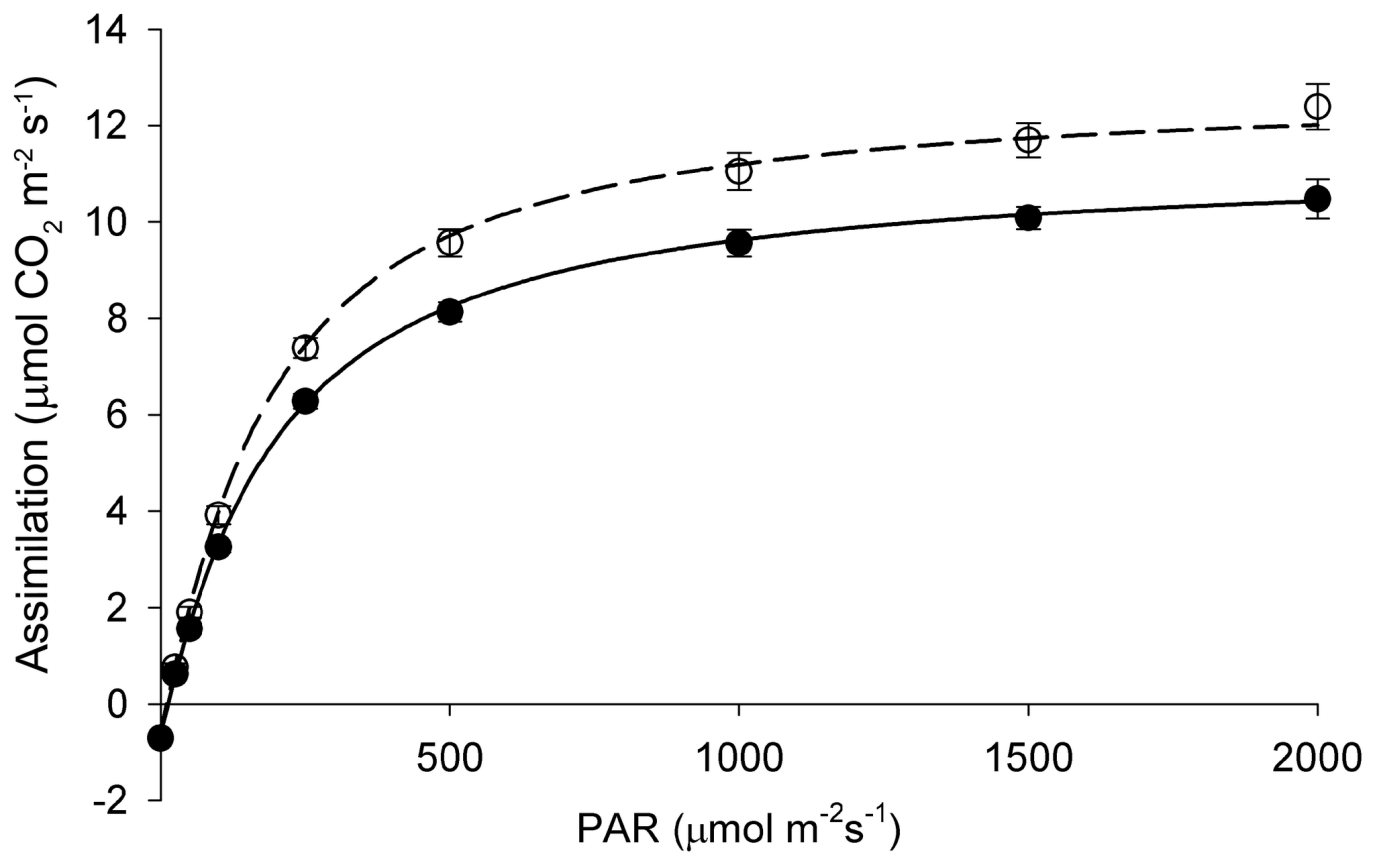
Light response curves for CO_2_ assimilation at 20^°^C in sun-grown green (*○*) and black (*●*) *Ophiopogon* *planiscapus* ‘Nigrescens’ leaves. Means + s.e., *n*=10.

Even though the black- and green-leafed *O* *. planiscapus* were near isogenic and grown adjacent to one another under full sun, they showed significant differences in their photosynthetic responses. Rates of CO_2_ assimilation were lower for the black than the green leaves at irradiances above approx. 400 µmol m^-2^ s^-1^ (Figure 2). Maximum rates of assimilation (A_max_) were 12% lower, while those of carboxylation (V_cmax_) and electron transport (J_max_) were each 15% lower in the black than in the green leaves (Table 1). In contrast, light saturation points (Q_sat_) were 21% higher for the black leaves (Table 1). None of the other measured parameters differed significantly between the colour morphs.

**Table 1:** Photosynthetic parameters from light- and CO_2_-response curves for black and green leaves of *Ophiopogon* *planiscapus* ‘Nigrescens’ at 20^°^C.

**Parameter**	**Black Leaves**	**Green Leaves**	***p***
*A*_max_ (µmol CO_2_ m^-2^ s^-1^)	12.0 ± 0.5	13.6 ± 0.6	0.04*
*g*_s max_ (mol m^-2^ s^-1^)	0.13 ± 0.02	0.15 ± 0.03	0.44 ns
*R*_d_ (µmol CO_2_ m^-2^ s^-1^)	-0.69 ± 0.06	-0.68 ± 0.06	0.44 ns
ϕ_a_ (mol mol^-1^)	0.054 ± 0.003	0.061 ± 0.004	0.08 ns
Θ	0.31 ± 0.05	0.42 ± 0.05	0.08 ns
*Q*_sat_ (µmol m^-2^ s^-1^)	1075 ± 97	849 ± 75	0.04*
*Q*_comp_ (µmol m^-2^ s^-1^)	12.8 ± 1.0	11.2 ± 0.8	0.11 ns
*V*_cmax_ (µmol m^-2^ s^-1^)	32.2 ± 1.6	37.8 ± 1.8	0.02*
*J*_max_(µmol m^-2^ s^-1^)	107 ± 4	126 ± 6	0.01*

A_max_, light-saturated CO_2_ assimilation; *g*_s max_, stomatal conductance at light saturation; R_d_, dark respiration; ϕ_a_, apparent quantum efficiency; Θ, convexity; *Q*_sat_, light saturation point; *Q*_comp_, light compensation point; *V*_cmax_, maximum rate of carboxylation; J_max_, electron transport rate at saturating c_i_.

**Figure 3:**
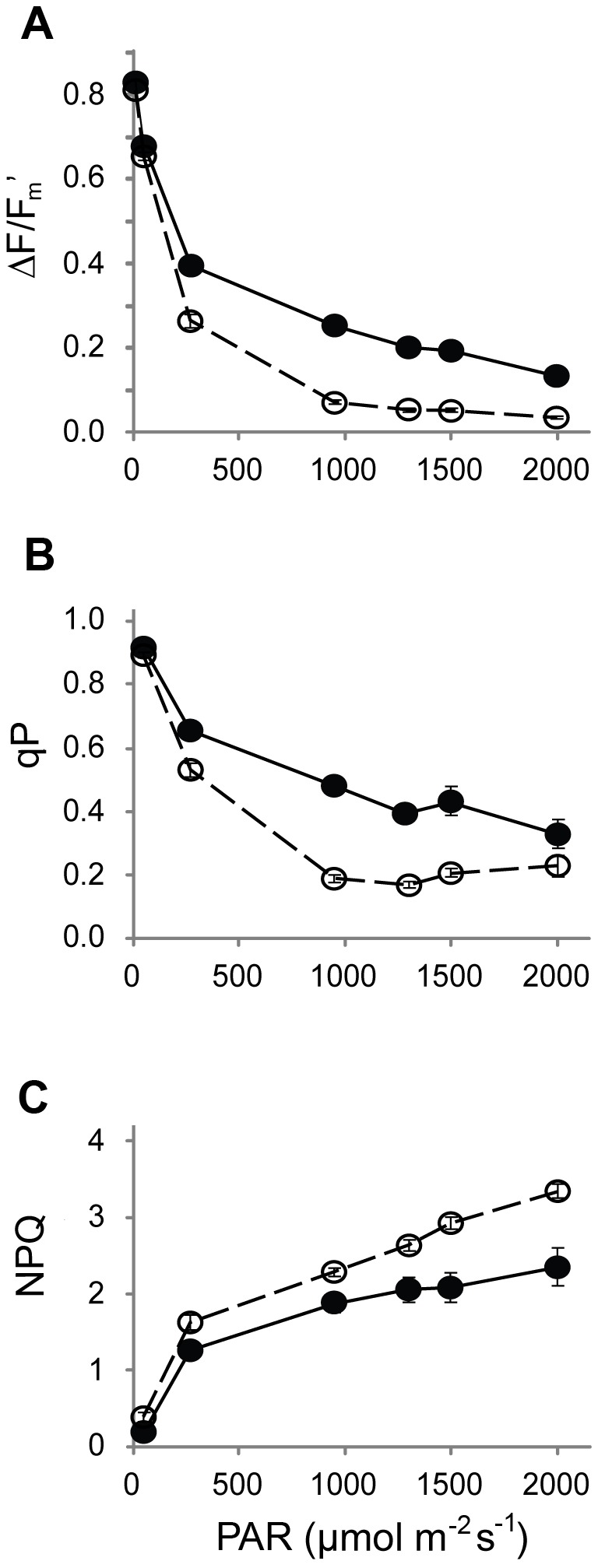
Light response curves for chlorophyll *a* fluorescence from adaxial surface of green (*○*) and black (*●*) leaves of sun-grown *Ophiopogon* *planiscapus* ‘Nigrescens’ at 20°C. Shown are (A) quantum efficiencies of PSII (ΔF/F_m_’), (B) photochemical quenching (qP), and (C) non-photochemical quenching (NPQ). Means ±s.e., *n*= 10. Error bars are smaller than symbols for most data points.

Maximum quantum efficiencies for PSII (F_v_/F_m_ values), as measured by chlorophyll *a* fluorescence in pre-dawn, dark-acclimated plants, approached 0.8 for leaves of both colours. When the leaves were illuminated with white light, quantum efficiencies of PSII (ΔF/F_m_’) decreased; the decline with increasing irradiance was more rapid and more extensive in the green than in the black morphs (ANOVA; *p* < 0.05; Figure 3A). Photochemical quenching (qP) also declined with increasing irradiance to about 1000 µmol m^-2^ s^-1^ (Figure 3B), and again the rate of decline was greater in the green leaves (*p* < 0.05). In contrast, non-photochemical quenching (NPQ) increased with irradiance, more in the green than in the black leaves (*p* < 0.05; Figure 3C).

### Responses to saturating light

**Figure 4:**
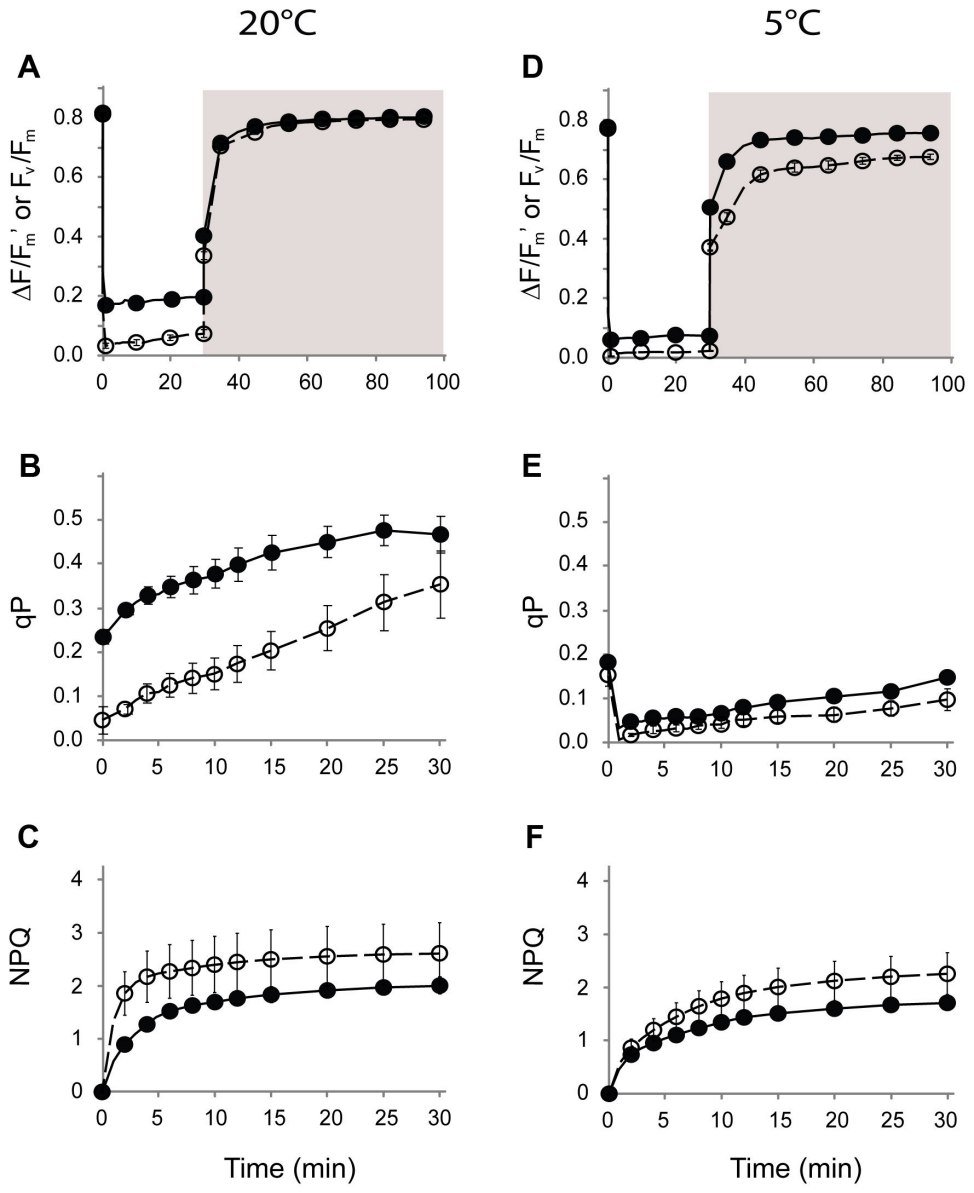
Chlorophyll a fluorescence parameters for green (○) and black (●) leaves of *Ophiopogon* *planiscapus* ‘Nigrescens’ at 20°C (A–C) or 5°C (D–F) in response to 30 min saturating white light at 1500 µmol photon m^-2^·s^-1^. Shown are quantum efficiencies of PSII (ΔF/F_m_’), photochemical quenching (qP), and non-photochemical quenching (NPQ) during light treatments, and recovery of ΔF/F_m_’ for 70 min following return of plants to darkness. Means + s.e., *n*=10.

In laboratory experiments at 20°C, a 30 min exposure to white light at 1500 µmol m^-2^ s^-1^ decreased the PSII quantum efficiencies of *O* *. planiscapus* leaves by more than 75% relative to their dark-adapted, maximum values. ΔF/F_m_’ values fell significantly more in the green than in the black leaves (*p* < 0.05; Figure 4A). When the plants were returned to darkness, quantum efficiencies recovered swiftly; F_v_/F_m_ values approached their original values within 30 min. During the light treatment, photochemical quenching (qP) increased, and was always far greater in black than in green leaves (*p* < 0.005; Figure 4B). Non photochemical quenching (NPQ) also increased, reaching a maximum at around 10 min into the light treatment (Figure 4C). NPQ values were on average larger for the green than the black leaves, but the difference was not statistically significant (*p* > 0.05).

To increase the probability of generating photo-oxidative stress, we repeated the high light treatment on fresh plants inside a refrigerated incubator at 5°C. All ΔF/Fm’ values fell close to zero under those conditions (Figure 4D). Quantum efficiencies of the black leaves recovered fully within 30 min of their return to darkness, but the green leaves recovered only up to 85% of their original values. This difference was maintained even after 8h in the dark (data not shown). The qP and NPQ values were lower under saturated light at 5°C than at 20°C. Differences in qP and NPQ between black and green leaves at 5°C were small (Figure 4E, F).

### Leaf temperatures

**Figure 5:**
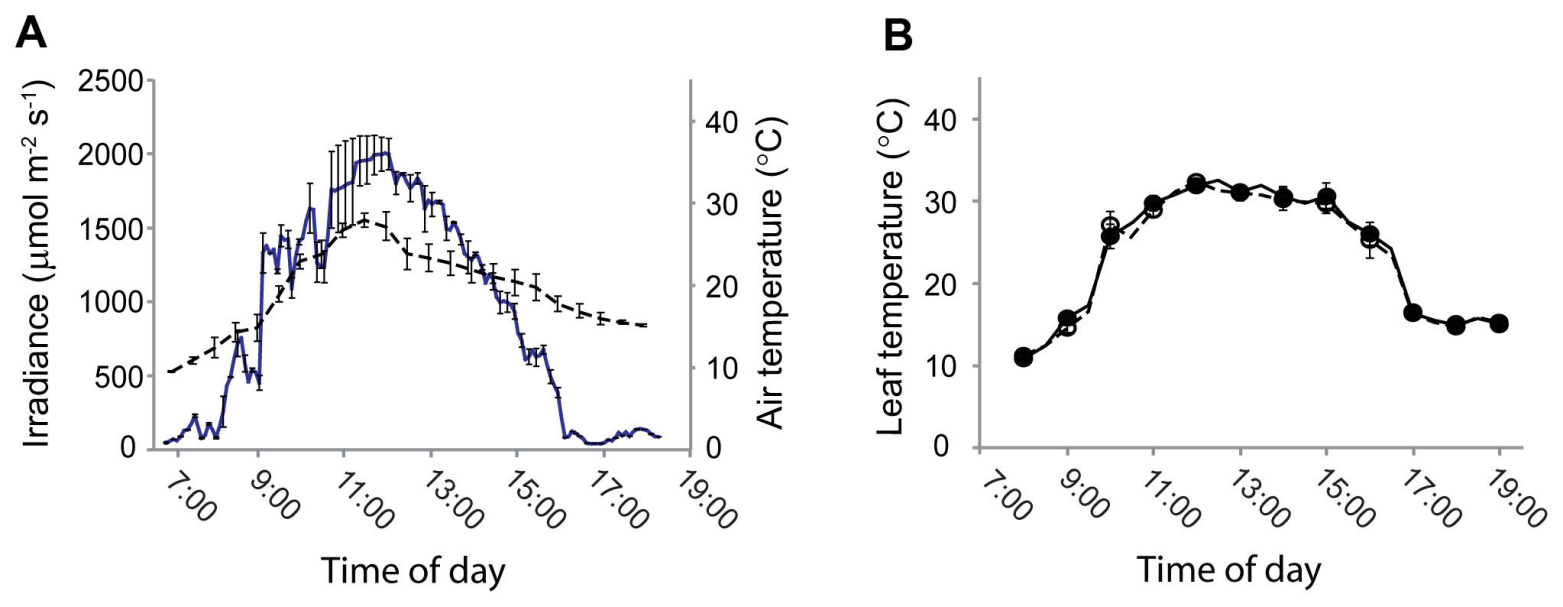
Diurnal changes in (A) irradiance (intact line) and air temperature (dashed line) at plant height, and (B) adaxial surface temperatures of green (○) and black (●) *Ophiopogon* *planiscapus* ‘Nigrescens’ leaves. Means + s.e., *n*=10.

To test the hypothesis that black pigmentation may lead to leaf warming, we monitored leaf and air temperatures at hourly intervals over the course of a summer’s day in Auckland, New Zealand. The day was for the most part cloudless, and air temperatures in the shade varied from dusk to dawn between 11°C and 27°C (Figure 5A). Natural irradiance incident on the leaves increased to approx 2000 µmol m^-2^s^-1^ PAR around midday, and remained above 1500 µmol m^-2^ s^-1^ for most of the day (Figure 5A). The adaxial surfaces of leaves were on average 3°C ± 0.5 warmer (*p* < 0.05) than the ambient temperature (Figure 5B). However, there were no significant differences in temperature between the leaves of the green and black phenotypes at any point during the day (*p* > 0.05; Figure 5B).

### Diurnal changes in quantum efficiency of PSII

**Figure 6:**
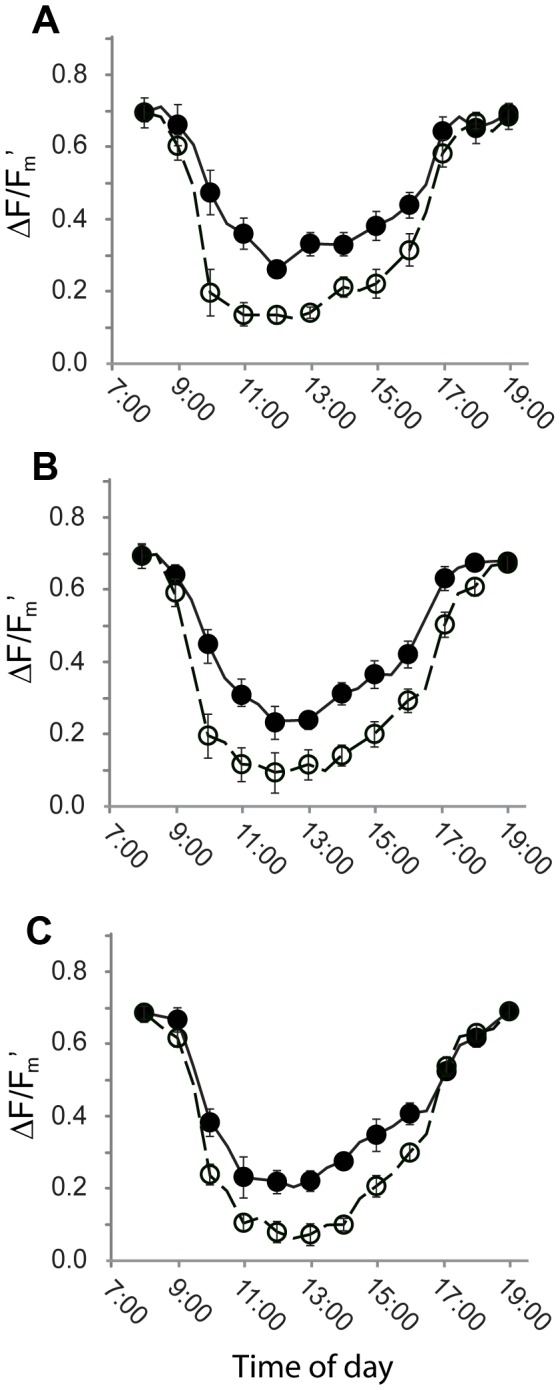
Diurnal changes in quantum efficiency of PSII (ΔF/F_m_’) for green (○) and black (●) leaves of sun-grown *Ophiopogon* *planiscapus* ‘Nigrescens’ under natural conditions shown in Figure 5A. Plants had acclimated to (A) 100%, (B) 40%, or (C) 10% full sunlight. Means + s.e., *n*=10.

To test whether the differences in PSII quantum efficiency recorded in the laboratory translated to an advantage in the field, diurnal changes in chlorophyll fluorescence were monitored outdoors under the conditions shown in Figure 5A. Hourly responses to full sunlight were compared for plants that had previously acclimated for 150 days under 10, 40, or 100% full sunlight. Substantial declines (*p* < 0.005) in ΔF/F_m_’ values were observed around midday for the leaves of both green and black *O* *. planiscapus* irrespective of their acclimation treatment (Figure 6A–C). ΔF/F_m_’ values invariably decreased more rapidly and to a greater degree (*p* < 0.005) in the green than in the black leaves. Between 10 am and 3 pm, ΔF/F_m_’ was, on average, 32% greater in the black leaves. Recovery was also swifter in the black than green leaves from all the acclimation treatments. Throughout the course of the day, NPQ values increased and then decreased, and qP values decreased and then increased, in pace with changes in irradiance (data not shown); there were no discernible differences in NPQ or qP between the two leaf colours (*p* > 0.05).

### Plant productivity

**Figure 7:**
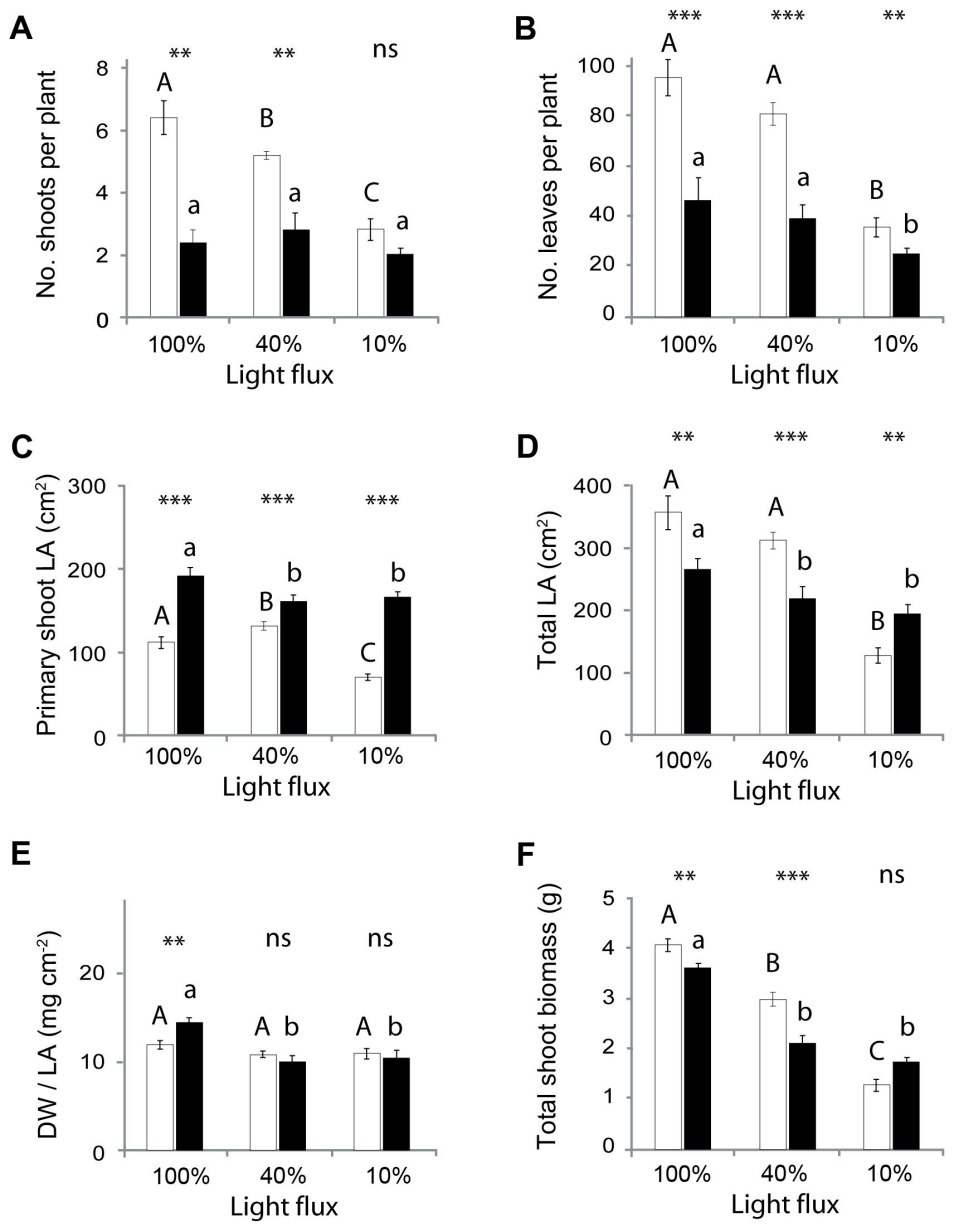
Productivity of green (open bars) and black (filled bars) shoots of *Ophiopogon* *planiscapus* ‘Nigrescens’ after 150 days in 10, 40, or 100% sunlight. (A) Total numbers of shoots per plant; (B) total numbers of leaves per plant; (C) total area of leaves borne on primary shoots; (D) total leaf area per plant; (E) specific leaf weights; (F) dry weights of shoots. Means ±s.e., *n*=10. Asterisks show significant differences between morphs within a treatment (*p<0.05; **p<0.005; *** *p*<0.001); letters above bars show significant differences across treatments (*p*<0.05). LA, leaf area; DW, dry weight.

The growth of plants was measured after 150 days under 10, 40, or 100% full sunlight. Although each pot initially held a single ‘primary’ shoot, in many cases these subsequently branched to produce several secondary shoots. The green-leafed plants branched more prolifically and produced many times more leaves than did the black ones, irrespective of their light environments (Figure 7A, B). The leaves borne on primary shoots were consistently larger for the black than the green plants (Figure 7C). Leaves on the secondary shoots tended to be shorter and more fibrous than those on the primary ones. Total leaf areas (primary and secondary shoots combined) were greater for the green phenotype when grown under high and medium light flux, but were greater for the black phenotype under the lowest light (Figure 7D). There were only small differences in specific leaf weight between black and green morphs (Figure 7E). Total shoot biomass was on average 11% greater for the green-than for the black-leafed plants grown under full sunlight, and 41% greater for green plants grown under 40% sunlight (Figure 7F).

Shading had significant effects on plant morphology. For both morphs, increased shading was associated with the production of fewer and smaller leaves, and a lower shoot biomass. Shading also reduced the numbers of branches on the green morphs, but not on the black morphs (Figure 7A–F).

### Leaf pigment concentrations

**Figure 8:**
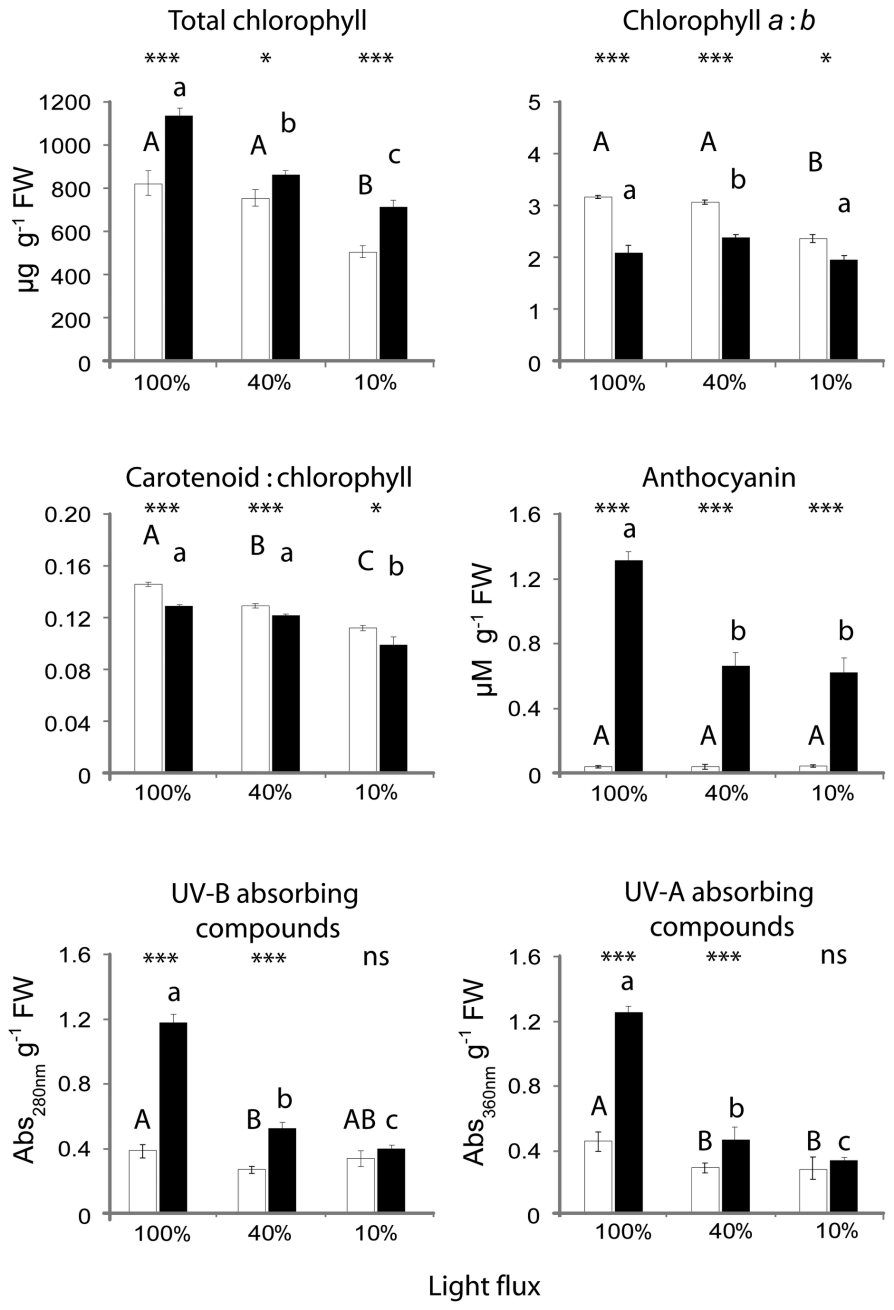
Pigment concentrations in the leaves of green (open bars) and black (filled bars) *Ophiopogon* *planiscapus* ‘Nigrescens’ grown under 100, 40 or 10% full sunlight. Means + s.e., *n*=10. Asterisks show significant differences within treatments (*p<0.05; **p<0.005; *** *P*<0.001); letters above bars show significant differences across treatments (*p*<0.05). FW, fresh weight; Abs, absorbance.

Irrespective of the light environment in which plants had grown, the black leaves had higher chlorophyll and anthocyanin contents than did the green leaves (Figure 8). In contrast, ratios of chlorophyll *a*: *b*, and of total carotenoids: total chlorophylls, were consistently lower in the black phenotype (Figure 8). UV-absorbing compounds, as estimated from the absorbances of methanolic extracts, were also more abundant in the black leaves, though only for those plants that had acclimated to 40% or 100% sunlight. The pigment concentrations in the black phenotype generally correlated to the amount of light received; all were greatest in those plants grown under full sunlight (Figure 8). The green leaves, too, held more chlorophyll and UV-A absorbing compounds per unit leaf mass when grown under full sunlight.

## Discussion

Black leafed *Ophiopogon* *planiscapus* assimilate less CO_2_ and accumulate less shoot biomass than do their green-leafed counterparts when grown under full sunlight. The 12% difference in maximum CO_2_ assimilation rates (Figure 2) corresponds closely to the 11% difference in shoot biomass between the black and green sun-grown plants (Figure 7F). The simplest explanation for these differences is that as a result of photoabatement by concentrated anthocyanin pigments in the mesophyll tissue, the chloroplasts in black leaves had acclimated to lower irradiances than had those in green leaves. Lower values of A_max_, V_cmax_, and J_max_ in black versus green leaves (Table 1) are consistent with the direction of differences typically found between shade- and sun-adapted leaves [29]. Similarly, the lower chlorophyll *a*: *b* ratios (Figure 8), and the reduced branching of black plants (Figure 7A) are classic responses to shading [29,30]. Analogous effects of anthocyanin pigments on the photophysiology of chloroplasts have been reported for red-leafed *Quercus* *coccifera* and *Quintinia* *serrata* [31,32].

A difference in productivity between black and green leaves was maintained when plants were grown in 40% sunlight, but was abolished under 10% sunlight (Figure 7F), even though anthocyanin concentrations in the two sets of black plants were similar (Figure 8). Anthocyanins attenuate predominantly green light [1], which excites chloroplasts in the abaxial mesophyll tissues when plants are exposed to strong white light. When exposed to dim white light, however, the effectiveness of green light as a driver of photosynthesis diminishes greatly [13,33]. Thus, it might be expected that the attenuation of green light by anthocyanins in the black leaves would only marginally affect net photosynthesis in the deeply shaded plants growing under 10% sunlight. This observation adds further support to a light-screening hypothesis for the effects of black leaf pigmentation.

Reductions in photosynthetic carbon assimilation may be partially offset by an enhanced protection against the effects of photoinhibitory stress. Under high light, quantum efficiencies for PSII (Ф_PSII_) were consistently greater for black than for green leaves, and under low light the efficiencies of the two phenotypes were at least comparable (Figure 3A). In addition, Ф_PSII_ was depressed considerably less in the black leaves following a photoinhibitory shock (Figure 4A, D). The advantage was especially pronounced at 5°C, after which Ф_PSII_ of black leaves recovered swiftly and completely, whereas the green leaves showed evidence of sustained photoinactivation. Moreover, the differences observed under controlled conditions in a laboratory translated into a real difference under natural sunlight. Depressions in photosynthetic efficiencies are commonplace during the sunniest part of the day [34]. In *O* *. planiscapus* , Ф_PSII_ of green leaves at midday in summer was depressed up to 1.5 times more than that of black leaves (Figure 6A–C). Both shade-grown and sun-grown plants benefited similarly from black pigmentation.

There are no obvious structural differences between leaves of the two colours that might explain the enhanced photoprotection of the black morphs [1]. Xanthophylls have long been known to assist in the dissipation of excess quantum energy from light-harvesting complexes [35]; however, ratios of total carotenoids to chlorophylls, which are an estimation of the dissipatory potential [36], were invariably lower in the black than the green leaves (Figure 8). Similarly, the chlorophyll fluorescence estimates of non-photochemical quenching, which correlate to thermal dissipation of excess energy [28], were almost always lower in the black leaves (Figures 3C & 4C, F). It is unlikely, therefore, that the carotenoids *per se* can explain the differences between black and green leaf photophysiology. There were also substantially higher concentrations of UV-A and UV–B absorbing compounds in the black than in the green leaves (Figure 8). These are likely to have assisted in the protection of chloroplast membranes and proteins from the adverse effects of UV exposure under the natural lighting experiment [37], but they cannot explain differences in photosynthesis between black and green leaves in the controlled laboratory experiments done in the absence of UV radiation.

Foliar anthocyanins have been found to diminish the frequency and severity of photoinhibition in many species [21,38,39,40,41,42,43,44,45,46,47,48], although exceptions have been reported. The unusually luxuriant concentrations of anthocyanins in black leaves of *O* *. planiscapus* ‘Nigrescens’ offer the most plausible explanation for their enhanced photoprotective capacities. Indeed, there is evidence that anthocyanin concentrations in *O* *. planiscapus* ‘Nigrescens’ are superabundant, at least in relation to photoprotective function. Plants grown under progressively higher light held increasingly more concentrated foliar anthocyanins (Figure 8), yet they were protected from light stress to a similar degree (Figure 6).

There are at least three hypotheses to explain how anthocyanins might assist photoprotection in this species. First, black leaves could be efficient absorbers of infrared radiation, resulting in leaf warming [49]; an elevation of leaf temperatures would reduce the propensity for photo-oxidative damage in colder climates. However, the temperatures of green and black leaves did not differ from one another (Figure 5B). Other workers have similarly failed to find temperature differences between anthocyanic (red) and green leaves [50,51].

Second, anthocyanins in the black leaves might protect the photosynthetic apparatus by scavenging reactive oxygen species (ROS) produced by chloroplasts and mitochondria [52]. An antioxidant function potentially explains why black-leafed plants, unlike the green plants, did not show chronic photoinhibition when subjected to a saturating light flux at 5°C (Figure 4D).

Third, differences in the photosynthetic responses between black and green leaves might result directly from light-screening; anthocyanins would absorb the energy of excess photons [17]. This was evidenced by greater qP values for black leaves in the light response curves (Figure 3B), suggesting a greater availability of the oxidized form of Q_A_, an electron acceptor of PSII. Photoabatement could also explain differences between black and green in the light response curve for Ф_PSII_ (Figure 3A).

Can the limitations to photosynthetic carbon assimilation account for the scarcity of black-leafed vascular plants in natural ecosystems? Many plant species accommodate between 10- and 20-fold differences in irradiance, leading to up to five-fold differences in the rate of light-saturated photosynthesis [53]. In comparison, the 12% difference in maximum CO_2_ assimilation rates between black and green *O* *. planiscapus* appears trivial. Of course, in addition to the lower area-based CO_2_ assimilation rate, the black morph also had a reduced total leaf area (Figure 7D), and therefore a lower potential for biomass accumulation. Furthermore, shoot: root biomass ratios would likely have been higher in the black plants, as is generally observed in shade-adapted plants. Thus, the difference in total biomass, including roots, could be larger between green and black plants than in shoot biomass alone. Nevertheless, both morphs appeared able to acclimate to irradiances of between 10% and 100% sunlight (Figure 7A–F), and under natural shade the black- and green-leafed plants appeared similarly matched (Figure 7F). There is, moreover, an apparent benefit of black pigmentation in terms of enhanced photoprotection, though this may not translate into a competitive advantage since the biosynthesis of anthocyanins incurs a metabolic cost, and since the green plants probably have an efficient, perhaps less costly, mechanism for the repair of photoinhibitory damage. Green- and black-leafed plants apparently thrive together in garden settings, even in dense plantings [12]. The dearth of black leafed vascular plants in natural populations, and their relative abundance among bryophytes and lichens, remains an intriguing enigma.
